# Species-specific responses drive browsing impacts on physiological and functional traits in *Quercus agrifolia* and *Umbellularia californica*

**DOI:** 10.1371/journal.pone.0287160

**Published:** 2024-07-24

**Authors:** Hugh E. Leonard, Mary Ciambrone, Jarmila Pittermann

**Affiliations:** Department of Ecology and Evolutionary Biology, University of California Santa Cruz, Santa Cruz, California, United States of America; University of Brescia: Universita degli Studi di Brescia, ITALY

## Abstract

Herbivory is a fundamental ecological force in the evolution of plant physiological, morphological, and chemical attributes. In this study, we explored how browsing pressure by local deer populations affected leaf form and function in two California native tree species, *Quercus agrifolia* (coast live oak) and *Umbellularia californica* (California bay laurel). Specifically, we investigated how leaf and stem vascular attributes differed between browsed and non-browsed zones of each species. Browsing significantly altered traits such as leaf to phloem ratios and leaf area, but we observed few meaningful differences in leaf and stem anatomy between browsed and non-browsed material. We discuss these results in the context of leaf and stem adaptations to herbivory and water use efficiency and explore future research considerations for investigating leaf and stem vascular trait development with herbivore presence.

## Introduction

Herbivory is a well-documented biological interaction influencing the ecology of natural systems [1–4]. Herbivores are ecosystem engineers because they can alter ecosystem structure and function through consumer activities and physical presence [5–7]. As primary consumers, herbivores impact ecosystem function by reducing aboveground biomass, altering resource allocation during competition with other plants, and reducing recruitment of new shoots and seedlings [8, 9]. A notable example is the re-introduction of wolves into Yellowstone National Park, which led to a decrease of elk in aspen thickets and a corresponding increase in new aspen shoot heights [8]. Furthermore, elk browsing and beaver presence in Yellowstone have been shown to have a complex interaction that determines annual growth and hydraulic properties in willows [10]. Under a different system, Chouinard and Filion found that white-tailed deer browsing significantly reduced sapling growth and impacted conifer forest regeneration [11]. These and other examples demonstrate how herbivore presence and density can transform the plant community’s physical structure and species composition, all of which can alter ecosystem function.

Herbivory can dramatically alter a plant’s morphometric traits at the level of individual plants. The most direct impacts arise from the alteration of resource allocation throughout the plant [12–14]. A notable example is the atypical growth that occurs when browsers remove the softer leaves and buds of the apical meristem region. Apical meristems produce the hormone auxin (indole-3-ascetic acid), which promotes terminal bud dominance. Browsing often removes the terminal buds, reducing auxin availability and as a result increasing lateral branching [15–17]. Thus through browsing, herbivores can stimulate plants to grow branches with shortened internode distances and compact foliage with less leaf tissue investment (thinner, smaller leaves) [18, 19]. Thinner and smaller leaves may result from reduced carbon resources available for growth, but this response may also be adaptive. For example, a reduction in leaf area and mass can be viewed as an adaptation to herbivory by reducing the carbon cost to the plant in the production of vulnerable leaves, in comparison to the upregulation of the more carbon-costly secondary compounds that reduce leaf palatability [3]. However, changes in leaf vascular structure and development may be the downstream consequence of reduced leaf area and carbon investment, and this may impact overall shoot water transport and photosynthetic capacity [20, but see also 21–24].

Previous work on *Ceanothus* has shown that long-term browsing reduces vessel density, stem hydraulic conductivity, and Huber values (sapwood to distal leaf area) [20]. Pittermann et al. observed a reduction in Huber values due to a reduction in leaf area for browsed stems, with browsing producing dense foliage with reduced leaf sizes and total leaf areas. Smaller leaves reduce the surface area available for gas exchange and can result in a decline of carbon uptake capacity, relative to bigger leaves. Plants can compensate for reduced leaf surface areas by producing higher stomatal densities, either through reduced guard cell size or reduced epidermal pavement cell size, but whether such alteration in tissues arises under browsing remains to be seen [22]. Specifically, it is unclear how browsing affects leaf cell and tissue structure, especially stomatal and epidermal cell traits critical to leaf water relations, and whether these changes could lead to permanent alterations in the structure and function of leaves and stems.

**Fig 1:**
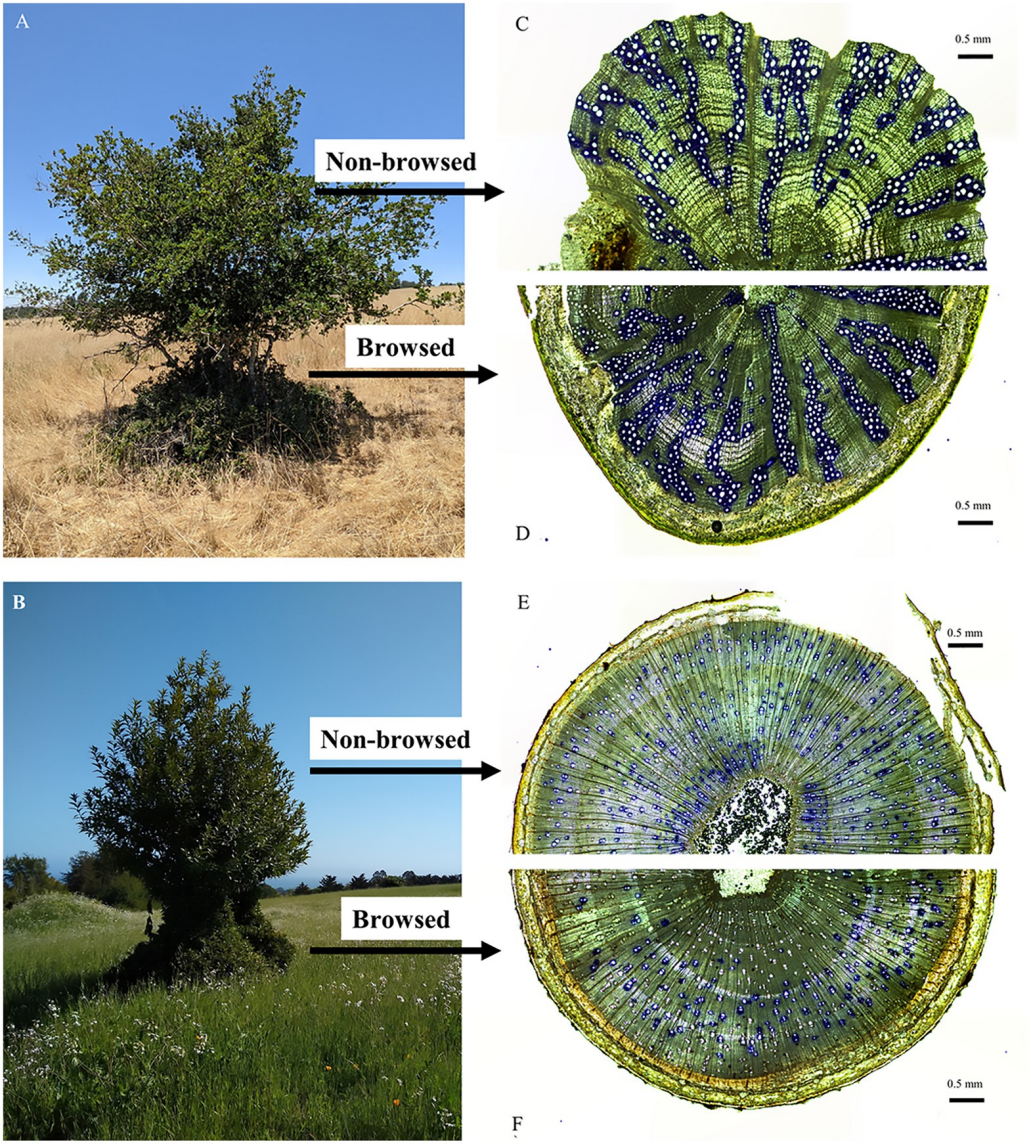
Morphology and stem anatomy of Q. agrifolia (A, C, D) and U. californica (B, E, F). Images A and B show tree morphology and topiary structure from deer browsing. Images C-F show functional xylem (stained blue) and general stem anatomy for non-browsed (C, E) and browsed (D, F) zones of each species.

Trees and shrubs experience intensive browsing at early life stages until growing high enough to escape this ’browse trap’ [6], resulting in effectively two growth habits at maturity: a browsed habit growing within the reach of herbivores and a non-browsed habit growing above the reach of herbivores (Fig 1A and 1B). Continued browsing maintains the browsed growth while allowing any growth above this browse trap to increase in stem and leaf dimensions. Such browsed and non-browsed growth systems within the same specimen provide the necessary natural experiment to test browsing effects on tissue development while maintaining other tree-specific ecological factors as constants.

To this end, we analyzed the effect of browsing activity of black-tailed deer (*Odocoileus hemionus columbianus*) on plant performance using leaf and vascular traits for two woody evergreen tree species, *Quercus agrifolia* (coast live oak), a ring-porous species, and *Umbellularia californica* (California bay laurel). We sought to understand how responses to herbivory can a) alter leaf and stem morphology and anatomy and b) what effect(s) these changes can have on water status within browsed zones of the plant. *Q*. *agrifolia* and *U*. *californica* specimens were selected growing within and along the edge of a grassland on the University of California, Santa Cruz campus and were exposed to the same deer browsing pressure. Significantly for this study, both tree species incurred deer browsing only between ground level and approximately 1.5 meters in height, above which the trees had no browsing impact. By selecting trees mature enough to have both browsed and non-browsed zones, we targeted our investigation on the direct impact browsing had on functional traits related to water conductance rather than the interaction of traits on development or the community ecology of species interactions on trait development [20]. Leaf traits including leaf mass per area (LMA), leaf vein density, stomatal and leaf epidermal cell density and size, and stomatal conductance were collected, as well as stem vascular traits including midday water potential, stem functional xylem area, xylem conduit size and density, and phloem tissue width in browsed and non-browsed zones for each species. We hypothesized that browsing would result in smaller leaves with smaller and fewer stomata, in coordination with reduced xylem and phloem vessel size and densities. Ultimately, we sought to clarify the mechanism of herbivory as a driver of vascular development, function, and the plants’ response to seasonal water deficit.

## Materials and methods

Following all applicable laws and university policies, collection of plant material was approved by the UCSC Upper Campus Reserve manager, Alex Jones (email: asjones@ucsc.edu). No permit number was required as we worked only on the university grounds. Additional information on collecting living and non-living samples for scientific research from the University of California, Santa Cruz can be found at https://ucsccampusreserve.ucsc.edu/about-us/collecting-policy.html.

### Site description

The study was executed at the University of California, Santa Cruz. The university is located on the southwestern edge of the Santa Cruz Mountains and experiences a Mediterranean climate with warm, dry summers and cool, moist winters. The average annual rainfall is 79 cm, and many summer days are characterized by afternoon and evening fog events [25]. *Q*. *agrifolia* specimens for this study occupied an open grassland area with full sun exposure. *U*. *californica* specimens were located within the same open grassland and were adjacent to a mixed evergreen forest edge. The surrounding habitat is primarily open and composed of low-growing, herbaceous species of grass and forbs (Fig 1A and 1B). To have a sufficient number of replicate trees per specimen, we selected two locations for our study, the Great Meadow and Porter Meadow, which occur at the same elevation and have the same physical and biological conditions (orientation, soil composition, biological community assemblage). The study sites lie approximately 900 meters apart and are separated by both mixed evergreen forest and campus buildings, but there is no restriction on deer movement either within or between sites. The campus lies adjacent to large amounts of protected land, which allows a local deer population to be supported year-round.

### Experimental design

*Q*. *agrifolia* and *U*. *californica* species were selected for this study from field observations of heavy browsing from the local black-tailed deer population, resulting in a strongly topiary growth habit (Fig 1A and 1B). The altered shape of these trees presented a natural experiment for investigating the physiological impact of browsing on leaf and stem tissues within the same tree. All samples and measurements were conducted on sun-exposed branches selected from a browsed height of less than 1.5 meters (hereafter called the browsed zone) and a non-browsed height of 1.5–2 meters (hereafter called the non-browsed zone) of each tree. As certain traits have been shown to change with large changes in tree height (such as leaf size and conductive vessel density), all samples were separated by no more than 1.5 meters in vertical height to minimize height effects [26]. Leaf and stem samples were collected in the field during late spring, from adult trees, selecting 2–3 year old branches with leaves undamaged by browsing and transported to the lab for processing. Midday water potentials and stomatal conductance measurements were collected in June and October 2021 from browsed and non-browsed zones to capture functional impacts on seasonal hydraulic capacity from browsing. All field data was collected between January 2020 and December 2021.

### Leaf traits

#### Collection of LMA

12 undamaged leaves were collected from both browsed and non-browsed zones of ten trees per species, for a total of 240 leaves per species (n = 120 for browsed and for non-browsed zones). Collected leaves were wrapped in moistened paper towels and placed in plastic bags for transport to the lab. Leaves were digitally scanned (HP Photosmart C6380 All-In-One Printer/Scanner; Hewlett-Packard, Palo Alto, CA, USA), and the images were processed using ImageJ software for leaf area (cm^2^; http://rsbweb.nih.gov/ij/index.html). After scanning, leaves were oven-dried for three days at 70 degrees Celsius and their dry mass was recorded [20]. Each leaf was weighed on a digital balance (grams; Acculab ALC series; Sartorius Group, Germany), avoiding air exposure until its mass was recorded. LMA was calculated as the ratio of dry leaf mass to leaf area [27].

#### Vein density

Three undamaged leaves were collected from browsed and non-browsed zones of ten Q. *agrifolia* and ten U. *californica* trees, resulting in 60 leaves per species (n = 30 for browsed and for non-browsed zones). Biopsy punches were used to generate three 4 mm diameter samples per leaf (RoyalTek biopsy punch; Surgical Design Inc, Lorton, VA, USA), selecting regions devoid of primary veins. Each leaf sample was treated with a solution of 1.0 M KOH in deionized water for pigment clearing. After 8 to 14 days, depending on the specific leaf samples and clearing rates, samples were removed and washed with deionized water before staining with toluidine blue. Stained leaf sections were mounted in glycerol, photographed under a 4X objective on a compound light microscope (Leica model DM6 B; Leica Microsystems, Germany), and analyzed for vein density using ImageJ software. Vein density for each species was calculated as the total vein length per leaf area averaged across each biopsy punch [28].

#### Stomatal traits

Stomatal measurements were made using one leaf per zone for each tree, utilizing the same three biopsy punches per leaf prepared for leaf vein densities (n = 10 leaves for browsed and for non-browsed zones per species). Stomatal density was captured under a 10X objective and measured as the number of stomata per square millimeter of leaf area. For stomatal anatomy measurements, stomata were photographed under 20X and 40X objectives. Each stoma within a biopsy punch was numbered, and the length and width of 5 haphazardly selected stomata per biopsy punch were measured (n = 150 stomata for browsed and for non-browsed zones per species). Stomatal size was approximated using stomatal length multiplied by stomatal width [29].

#### Epidermal cell traits

Epidermal measurements, specifically pavement cells [30], were made using one leaf per zone for each tree, utilizing the same three biopsy punches per leaf prepared for leaf vein densities (n = 10 leaves for browsed and 10 non-browsed zones per species). Five pavement cells per biopsy punch region were imaged under 20X and 40X microscope objectives (n = 150 for browsed and for non-browsed zones per species). Pavement cells were selected by outlining a leaf region and selecting cells from four corners of the viewing field and one cell from the center. Pavement cell area was measured manually by tracing the outline of each cell using ImageJ software and recording the area enclosed.

#### Stomatal conductance and midday water potential

To assess differences in water status between browsed and non-browsed regions, mid-day water potential (Ψ_mid_) and stomatal conductance were collected from six *Q*. *agrifolia* and *U*. *californica* trees (n = 6 trees per species). Measurements were collected during June of 2021 prior to the onset of the summer dry period and again in October 2021 at the end of the summer dry season. Measurements were obtained from exposed leaves of at least one year of age and collected between 10 am and 3 pm to capture maximum leaf transpiration and xylem water tension. Stomatal conductance was collected using a portable porometer (SC-1 leaf porometer; Decagon Devices, Pullman, WA, USA) on three separate leaves (n = 18 measurements for browsed and non-browsed zones, per species), and Ψ_mid_ was collected on the same branchlets using a Scholander-style pressure chamber (PMS Instruments, Corvallis, OR, USA) [31].

### Stem traits

#### Stem selection and age

Stem traits were obtained from six trees per species, with one branch selected from the browsed zone and one from the non-browsed zone of each tree (n = 6 branches for browsed and for non-browsed zones, per species). Branch length and internode distance varied between browsed and non-browsed zones due to deer browsing, therefore direct observation could not be reliably used to determine branch age. To ensure that age was not a confounding factor in stem structure and function, each branch was confirmed to be between two and four years of age based on the number of growth rings present after cutting. Branches were covered in dark plastic bags before dawn for a minimum of 15 minutes to allow water potential within the xylem water column to equilibrate to the atmospheric conditions and reduce tension on the xylem water column [20, 32]. Each branch was then cut and quickly submerged under water before cutting an additional 3cm of the stem to minimize the introduction of air into conductive xylem vessels. Sample branches were then transported within three hours to the lab for processing, with the cut ends kept submerged to avoid introducing air into xylem vessels and embolism formation.

#### Functional xylem

To identify functional xylem vessels, protocols were followed as outlined by Jacobsen et al. [33], imposing a negative pressure of 2kPa on 12 cm stem portions to draw crystal violet stain into the functional vessels of the stem. This pressure was applied for five minutes, after which segments were removed from the vacuum system and deionized water was used to flush excess stain from the segments to prevent false positives in conductive vessel identification. All segments were then frozen immediately to avoid diffusion of stain into non-conductive tissues, and frozen segments were used to generate a transverse cross section from the center of each segment using a sledge microtome. Freezing the samples avoided excessive sample fracturing during sectioning.

The prepared cross sections of each stem were mounted on slides with glycerol and photographed under 4X objective (n = 6 cross sections for browsed and for non-browsed zones, per species). To record conductive vessel density, rather than count all vessels within a cross section, cross sections were partitioned using ImageJ into three 33 degree wedges (± 1 degree) extending from the pith to the bark and arranged equidistantly around the cross section when possible. All conductive vessels were counted within each wedge, and the longest and shortest lumen diameters for each vessel were recorded. All hydraulic diameters from each wedge were averaged to find the mean vessel hydraulic diameter for the stem sampled, and these values were then compared within each species for differences between browsed and non-browsed zones. [34].

#### Phloem width

Phloem data were collected on the same cross sections used for xylem analysis (n = 6 cross sections for browsed and for non-browsed zones, per species). Phloem width was determined from the average of four width measurements collected from each cross section and separated by 90 degrees when possible. Phloem area was calculated as the difference in stem area minus bark and xylem tissue, and measurements were made using the assumption of a circular area (πr^2^).

### Data analysis

All statistical analysis was conducted using R 4.2.2 [35]. We recorded trait values across 6–10 replicate trees to account for individual variation within each species. We averaged the data on a per trait basis to obtain a species-level response in hydraulic function to browsing pressure. Since we were investigating the response of each species to browsing, rather than an interspecific analysis and comparison of trait response, we were able to conduct the majority of our statistical analysis for each species in a simplified control-treatment model and not have to account for interspecific variation in traits that require more advanced modeling We used Welch’s t-tests to compare trait means between browsed and non-browsed zones within our replicate specimens because of differences in trait variance between zones. We also used paired t-tests to compare seasonal differences in midday water potentials and stomatal conductance rates for each species within each zone. For analysis of correlated traits, we used the cor() package in R to create a correlation matrix using Pearson coefficients and visualized these results using the corrplot package v0.92 [36] (S1–S4 Figs). To obtain correlation coefficients between leaf and vascular traits, we used a subset of our data that included leaf and stem samples from the same trees (n = 6 for *Q*. *agrifolia* and n = 4 for *U*. *californica*). To assess whether a difference in the coordination of leaf cell traits, specifically leaf pavement cell size on stomatal density, occurred under browsing, we used the lm() function in R to create linear regression models for browsed and non-browsed zones within each species (S1 Table). All results were considered significant if p < 0.05.

## Results

### Leaf morphometric and anatomical traits

**Fig 2:**
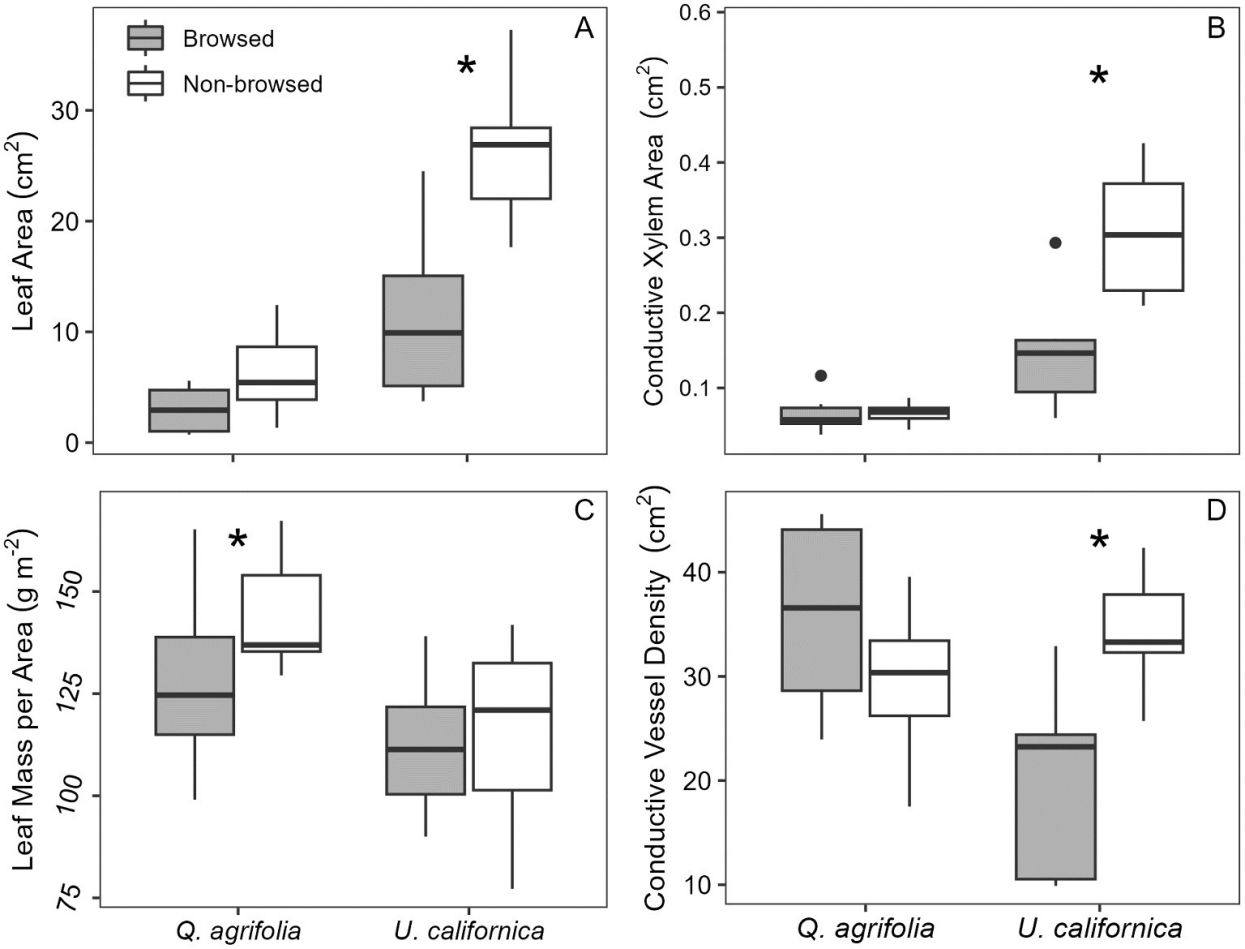
Comparison of browsed and non-browsed leaf traits. Boxplots of browsed and non-browsed (A) leaf area, (B) conductive xylem area, (C) leaf mass per area (LMA), and (D) conductive vessel density. In panel A, *U*. *californica* showed a significantly larger leaf area for non-browsed zones (p = 0.0061), while in panel C *Q*. *agrifolia* had a significantly greater LMA for non-browsed zones compared to browsed zones (p = 0.049). Panels B and D, respectively, indicate *U*. *californica* browsed zones contained significantly less conductive xylem (p = 0.0099) and had a lower density of conductive vessels (p = 0.031) compared to non-browsed zones. Asterisks indicate significant differences (p < 0.05) between browsed and non-browsed zones for each species.

Leaves were generally smaller and had lower LMA in browsed zones, although the differences were inconsistent between species (Table 1, Fig 2). For example, leaves were approximately 57% smaller (p = 0.0061) in browsed *U*. *californica* compared to non-browsed zones. However, LMA remained largely invariable in browsed and non-browsed zones. In oak, by contrast, leaf mass per area was 11% lower (Table 1, p = 0.035) in browsed zones while leaf area was not statistically different from non-browsed zones, suggesting that continual browsing reduces the available resources needed to construct robust new leaves. (Fig 2B). Taken together, this suggests that both leaf size and LMA vary as a function of species-specific responses.

**Table 1:** Comparison of physiological traits between browsed and non-browsed zones.

***Q*. *agrifolia***			
**Trait**	**Browsed**	**Non-browsed**	**p-value**
Internode Length (cm)	2.28 ± 0.76	10.46 ± 2.95	**7.63e-4**
Branch Length (cm)	30.4 ± 16.64	55.15 ± 11.11	**0.0148**
Leaf Mass per Area (LMA) (g m^-2^)	128.48 ± 19.33	144.31 ± 13.36	**0.049**
Leaf Area (cm^2^)	299.021 ± 219.70	628.46 ± 406.13	0.12
Stomatal Size (um^2^)	593.94 ± 75.98	635.38 ± 43.66	0.157
Huber Value (functional xylem/leaf area)	3.33e-04 ± 1.92e-04	1.77e-04 ± 1.71e-04	0.17
Pavement Cell Size (um^2^)	215.72 ± 42.29	191.14 ± 38.02	0.189
Mean Phloem Width (cm)	0.035 ± 7.09e-03	0.040 ± 2.72e-03	0.218
Conductive Vessel Density (m^-2^)	3.59e+07 ± 9.30e+06	2.95e+07 ± 7.61e+06	0.225
Vein Density (per cm^2^)	819 ± 186	947 ± 266	0.23
Conductive Mean Conduit Diameter (um)	33.84 ± 4.61	37.01 ± 4.76	0.268
Conductive Hydraulic Diameter (um)	32.50 ± 4.51	35.58 ± 4.76	0.277
Stomatal Density (cm^-2^)	41950 ± 8247	46180 ± 10169	0.321
Phloem to Xylem Width Ratio	0.17 ± 0.040	0.18 ± 0.026	0.467
Stem Areas (cm^2^)	0.54 ± 0.14	0.56 ± 0.14	0.779
Stomatal Conductance (mmol m^-2^ s^-1^)	474.96 ± 58.38	466.47 ± 64.72	0.787
Midday Water Potential (MPa)	1.23 ± 0.56	1.28 ± 0.36	0.826
Conductive Xylem to Stem Area Ratio	0.12 ± 0.033	0.12 ± 0.048	0.955
Phloem Area (cm^2^)	0.056 ± 0.010	0.056 ± 0.013	0.973
Mean Xylem Width (cm)	0.22 ± 0.052	0.22 ± 0.019	0.992
Conductive Xylem area (cm^2^)	0.067 ± 0.028	0.066 ± 0.015	0.9995
***U*. *californica***			
**Trait**	**Browsed**	**Non-browsed**	**p-value**
Internode Length (cm)	3.26 ± 2.28	25.04 ± 7.73	**6.31e-4**
Stem Phloem to Leaf Area Ratio	0.33 ± 0.11	0.13 ± 0.07	**0.0046**
Leaf Area (cm^2^)	1139.44 ± 796.59	2633.83 ± 684.54	**0.00607**
Conductive Xylem area (cm^2^)	0.15 ± 0.083	0.31 ± 0.090	**0.0099**
Branch Length (cm)	47.10 ± 21.29	91.53 ± 27.48	**0.0115**
Conductive Vessel Density (m^-2^)	2.02e+07 ± 9.85e+06	3.43e+07± 6.24e+06	**0.0314**
Stem Areas (cm^2^)	0.41 ± 0.095	0.62 ± 0.20	0.0582
Conductive Xylem to Stem Area Ratio	0.35 ± 0.15	0.50 ± 0.045	0.0582
Mean Xylem Width (cm)	0.21 ± 0.040	0.25 ± 0.033	0.0741
Phloem to Xylem Width Ratio	0.10 ± 0.032	0.076 ± 0.014	0.146
Huber Value (functional xylem/leaf area)	1.48e-04 ± 4.20e-05	1.18e-04 ± 2.30e-05	0.158
Conductive Hydraulic Diameter (um)	27.26 ± 5.83	31.07 ± 0.80	0.218
Conductive Mean Conduit Diameter (um)	28.86 ± 6.15	32.76 ± 0.76	0.23
Midday Water Potential (MPa)	1.12 ± 0.37	0.98 ± 0.37	0.419
Pavement Cell Size (um^2^)	534.72 ± 153.45	490.79 ± 116.12	0.48
Leaf Mass per Area (LMA) (g m^-2^)	111.08 ± 15.99	115.92 ± 21.57	0.576
Phloem Area (cm^2^)	0.028 ± 0.011	0.031 ± 0.012	0.604
Stomatal Size (um^2^)	419.31 ± 71.57	405.11 ± 63.68	0.645
Stomatal Density (cm^-2^)	21391 ± 6648	20397 ± 4879	0.708
Mean Phloem Width (cm)	0.02 ± 6.22e-03	0.019 ± 4.80e-03	0.712
Stomatal Conductance (mmol m^-2^ s^-1^)	179.09 ± 61.96	168.80 ± 63.42	0.718
Vein Density (per cm^2^)	458 ± 140	434 ± 159	0.725

Trait means, ± standard deviation, with t-test outputs comparing browsed and non-browsed leaf and stem tissue for *Q*. *agrifolia* and *U*. *californica*.

**Fig 3:**
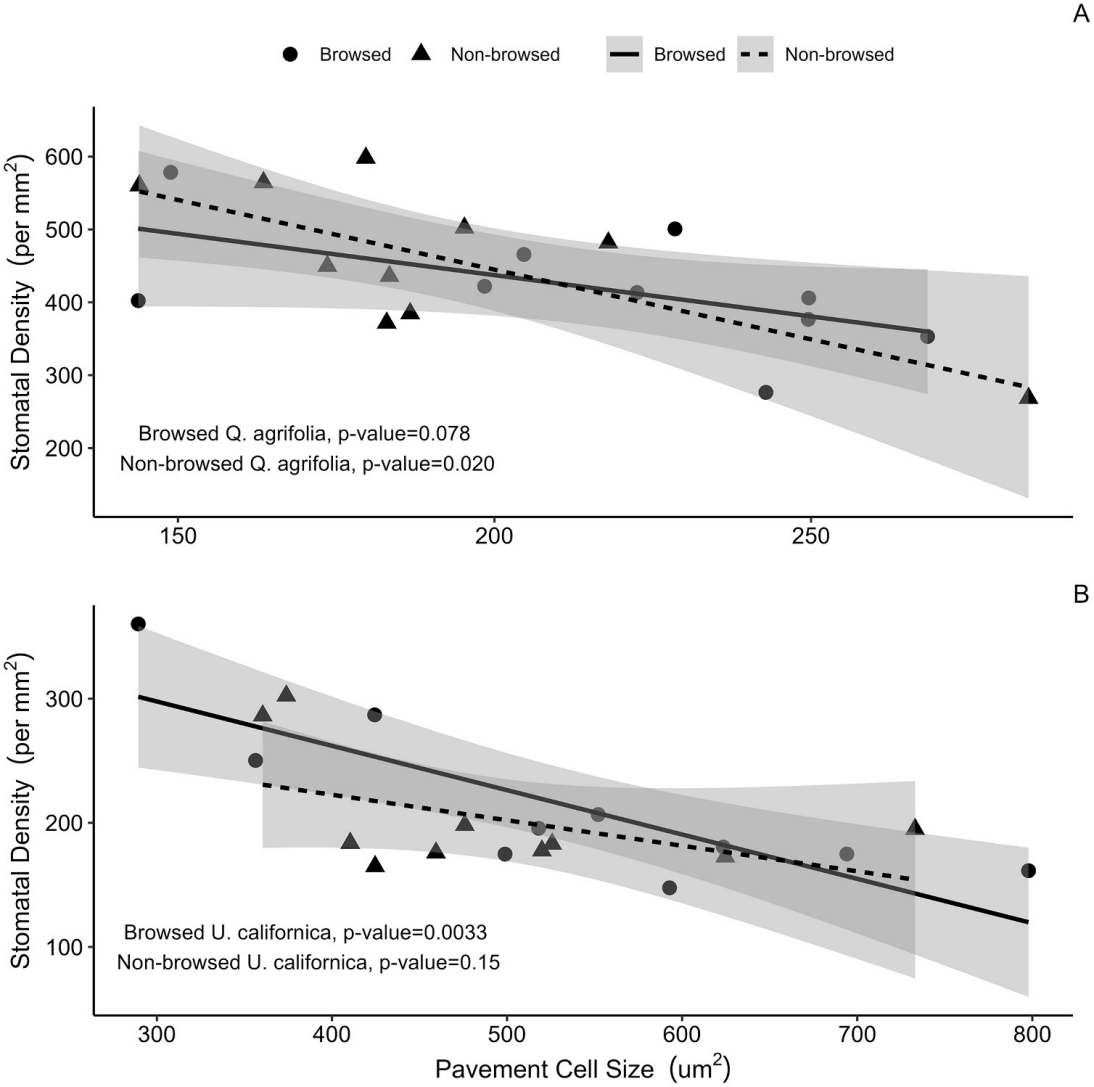
Leaf stomata and pavement cell coordination across browsed and non-browsed zones. Linear regression of browsed and non-browsed zones of Q. *agrifolia* (A) and *U*. *californica* (B). Both species showed a negative correlation between increasing stomatal density and pavement cell size, but this trend was only significant in non-browsed zones of *Q*. *agrifolia* (p = 0.020) and browsed zones of *U*. *californica* (p = 0.033).

Vein densities for *Q*. *agrifolia* and *U*. *californica* were similar for leaves from browsed and non-browsed zones (Table 1). Likewise, the size and density of both species’ leaf stomatal and pavement cells did not differ between browsed and non-browsed zones. However, we did notice interesting relationships between leaf traits in both species. For example, an increase in pavement cell size was associated with lower stomatal density in both species (Fig 3A and 3B). However, this was observed in leaves from non-browsed zones in oak (p = 0.020) and browsed zones in *U*. *californica* (p = 0.0033). This suggests that leaf development in response to herbivory is inconsistent and can vary in different species.

Taken together, leaf trait responses to herbivory were variable across each species. While leaves from browsed zones were, on average, half the size of the non-browsed zone leaves, leaf tissue investment varied by species, with lower LMA in the browsed zones of oak and no difference between *U*. *californica* browsed and non-browsed zones. Anatomical traits related to pavement cell size and density, stomatal size and density, and vein density were also unchanged in browsed zones relative to non-browsed zones. However, the relationships between these traits varied at a species-specific level to herbivory.

### Water potential and stomatal conductance

**Fig 4:**
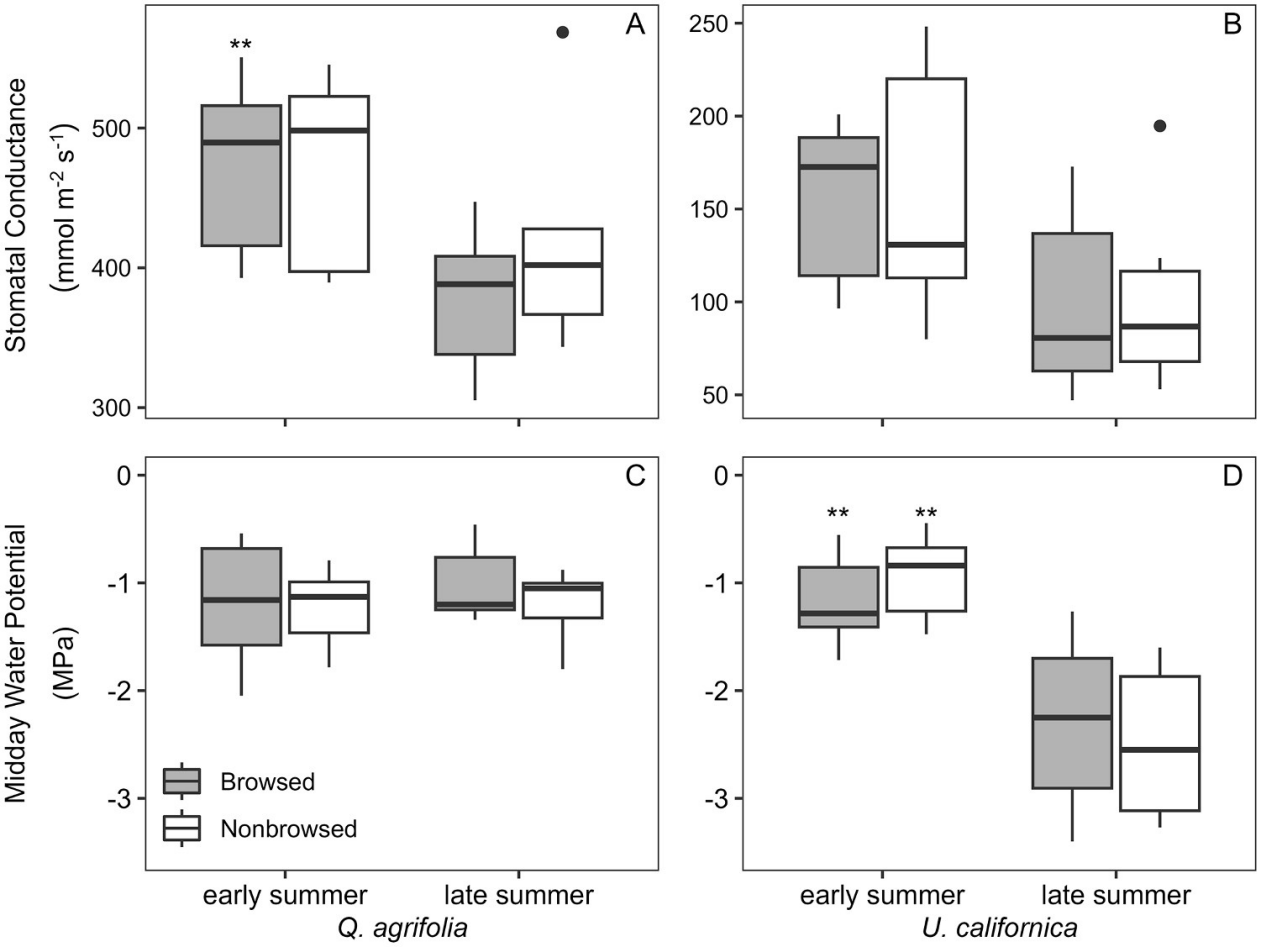
Seasonal midday water potential and stomatal conductance for *Q*. *agrifolia* and *U*. *californica*. Trait means, ± standard deviation, with (**) representing highly significant changes in trait values from early to late summer (p < 0.01). Both *Q*. *agrifolia* (A, C) and *U*. *californica* (B, D) showed no significant differences between browsed and non-browsed tissues. In panel A *Q*. *agrifolia* did show seasonal differences in stomatal conductance in browsed zones (p = 0.00965), while in panel D *U*. *californica* showed seasonal differences in mid-day water potential for both browsed and non-browsed zones (p = 0.00913 and 0.00198, respectively).

Neither species exhibited differences in Ψ_mid_ or stomatal conductance in browsed and non-browsed zones during the sampling period, although both midday water potential and stomatal conductance were lower toward the end of the growing season (Fig 4). This suggests that the water relations of the trees achieved some equilibrium between the browsed and non-browsed regions. However, *U*. *californica*’s water potential was significantly lower than *Q*. *agrifolia* at the end of the season; this species may not be as deep-rooted as the *Q*. *agrifolia*.

### Stem morphometric and anatomical traits

Browsed zones extended 1.5 meters from the tree’s base, with leaves above that height presumably being out of reach for the deer. The perpetual nibbling created an obvious topiary architecture due to reduced growth at the base of the trees. Consequently, browsed branch length was substantially shorter in *Q*. *agrifolia* stems than in non-browsed stems (Table 1; p = 0.0148), and a similar outcome was observed in U. californica (p = 0.0115). In addition to shorter branches, branch internode length was significantly shorter for browsed branches in *Q*. *agrifolia* (p = 7.63e-4) and in *U*. *californica* (Table 1; p = 6.31e-4), producing a condensed canopy of foliage under browsing pressure.

### Xylem anatomy and functional traits

**Fig 5:**
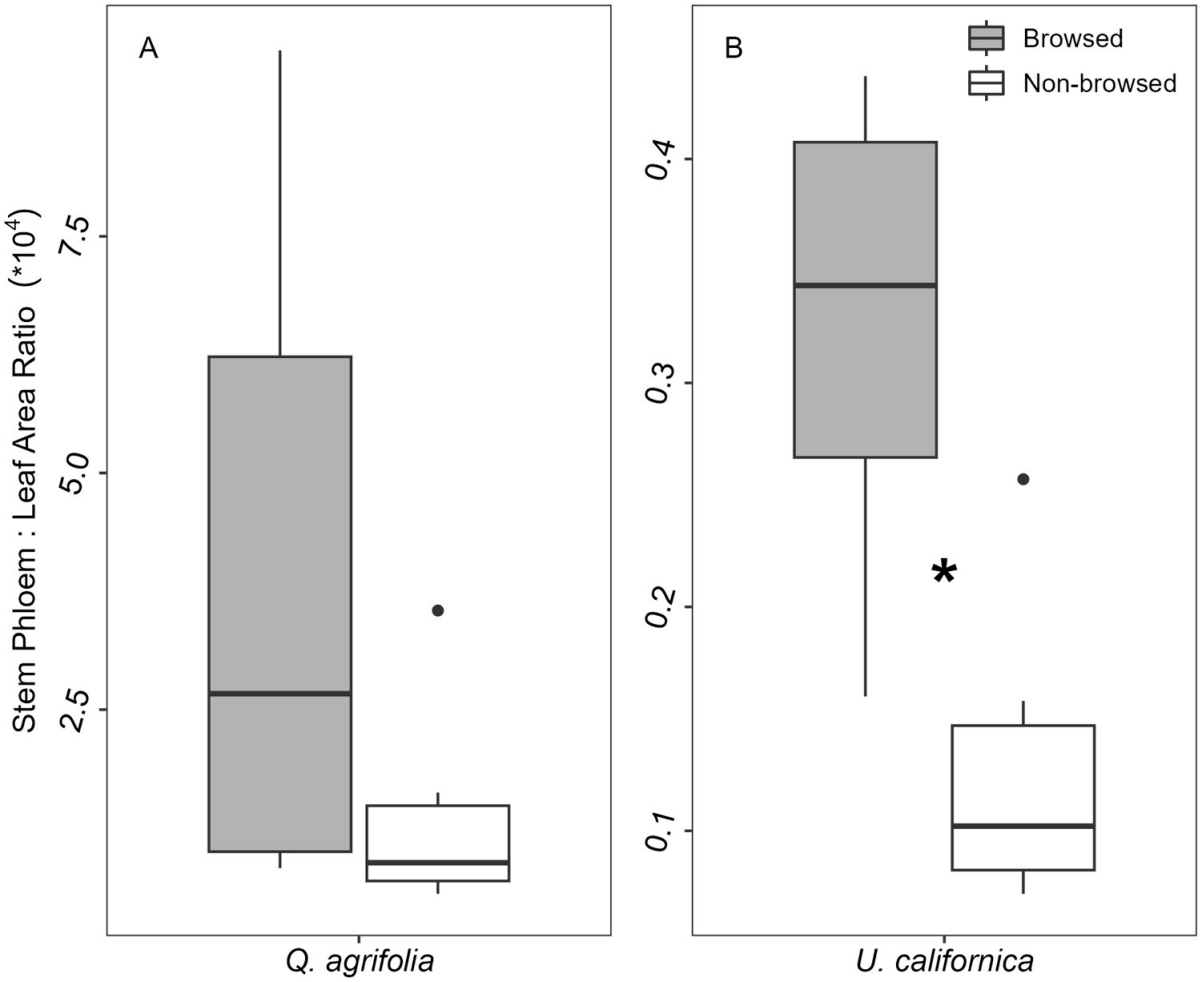
Ratio of stem phloem area to leaf area for *Q*. *agrifolia* and *U*. *californica*. Browsed zones of *U*. *californica* had a significantly higher ratio of stem phloem area to leaf area than non-browsed zones (p = 0.0046) due to the reduced total leaf area in browsed zones. Significant differences (p < 0.05) are represented by asterisks.

In *Q*. *agrifolia*, no traits related to xylem or phloem function appeared to differ significantly between browsed and non-browsed stems. By contrast, *U*. *californica* showed a significant 48% reduction in conductive xylem area in stems from the browsed zone (Fig 2B, Table 1; p = 0.0099). Due to the concurrent reduction in both leaf and conductive xylem areas for browsed stems, *U*. *californica* Huber Values were similar for both browsed and non-browsed stems (1.48e-04 ± 4.20e-05 browsed and 1.18e-04 ± 2.30e-05 non-browsed, p = 0.158). However, the decrease in leaf area did not lead to a decrease in stem phloem area in browsed stems (Table 1, Fig 5; p = 0.604), suggesting a disconnect between phloem production and leaf area trait function.

## Discussion

Within this study, we sought to answer questions concerning browsing impacts on the development and function of leaf and stem vascular traits, particularly how browsing may alter tissue form and function within an individual tree. The results suggest that browsing uniquely impacts each species’ leaf and stem vascular function. Taken together, these results suggest that species life history factors into the herbivory response in ways that are not easily predicted.

### Leaf traits

Our initial hypothesis was that browsing would lead to a decreased investment in leaves growing in browsed zones of both species, with browsed zones having fewer leaves with lower LMA compared to leaves from non-browsed zones. Interestingly, we observed that each trait, leaf area and LMA, became altered independently in each species; leaf LMA declined significantly in browsed zones of *Q*. *agrifolia* but not in *U*. *californica*, while mean leaf area declined in browsed zones of *U*. *californica* but not in browsed *Q*. *agrifolia*. While we did not directly investigate the mechanism(s) leading to these changes, leaf size and LMA differences could result from browsing effects and the alteration of auxin production in terminal buds. Research has shown that repeated wounding increases auxin production within the surrounding plant tissues [37]. Higher auxin levels signal the release of jasmonic acid hormones (JAs) for defense. Increased JA levels have been correlated with stunted growth patterns in plants, primarily in the form of reduced tissue cell numbers [37, 38]. Potential ramifications from the reduction in leaf area and thickness include changes in water transport throughout the leaf and leaf water loss, although we did not observe this in our species. It is possible that the alteration in leaf traits we observed are compensated for, perhaps through altering stomatal pore size, to maintain an optimal functionality.

In other aspects of leaf structure, the effects of herbivory become more nuanced. We expected herbivory to produce "cheaper" leaves with reduced carbon allocation to browsed zones, resulting in lower vein density and a corresponding reduction in stomatal density. Instead, we found that both species’ vein and stomatal density were invariable across browsed and non-browsed leaves. This suggests that leaf vascular structure is not as strongly impacted by herbivory as we predicted based on earlier work [20]. Additionally, there was no difference in stomatal conductance rates between zones, which correlates with the lack of stomatal size or density changes. Past research has shown that simulated defoliation can lead to nearly doubling stomatal conductance rates for defoliated stems compared to control treatments, likely due to an increased root-to-shoot ratio [32, but see 39]. In our study, using mature trees that maintained intact foliage after browsing may have obscured the shifts in stomatal conductance observed in previous studies on smaller individuals. However, the lack of stomatal density or regulation differences between browsed and non-browsed zones still raises the possibility that browsed zones may experience a shift in photosynthetic capacity compared to non-browsed zones. While beyond the scope of this study, future research on the long-term impact of herbivory on photosynthetic rate should be considered to assess whether this decline in photosynthetic capacity occurs and, if it does, to what extent it impacts plant growth and performance.

Variation in epidermal pavement and stomatal cell size was of particular interest in our study, as research has shown that epidermal cell size impacts leaf water loss [22]. Our initial hypothesis was that herbivory would reduce stomatal size and density due to reduced demand for water in response to lower leaf area. We further speculated that smaller pavement cells would arise as a consequence of developmental coordination. However, browsing did not affect either species’ pavement or stomatal cell sizes or densities. By contrast, we did observe coordination between increasing pavement cell size and decreasing stomatal density, but only in leaves collected from non-browsed zones of *Q*. *agrifolia* and browsed zones of *U*. *californica*. It is unclear why browsing would lead to opposite effects in coordination between these two traits, but possible explanations include species-specific reallocation of resources in browsed leaves or alteration of hormone production and regulation in developing leaf tissue when browsing occurs [40, 41].

### Vascular traits

As with leaf traits, each species exhibited specific trait responses to browsing. In *Q*. *agrifolia*, the browsed and non-browsed tissues showed no significant differences in the vascular traits investigated. While an apparent reduction in internode length was observed in the field and for sampled branches, this difference did not alter vascular traits within the stem. In *U*. *californica*, however, several traits did show a significant difference between treatment types.

For *U*. *californica*, conductive stem vessel densities and conductive xylem areas were significantly lower in browsed zones than in non-browsed. This is an intuitive result of browsing pressure, as reduced leaf area leads to reduced demand for vascular supply. Secondly, reduced vessel densities may reflect continuous vessel breakage during browsing, leading to air entry and embolism formation [42]. It is interesting to note the difference with *Q*. *agrifolia*, as similar browsing damage to stems appeared across both species but only *U*. *californica* responded. This species-specific response could be attributed to the differences in the vascular arrangement within stems, with *Q*. *agrifolia* exhibiting a “clustering” of vessels surrounded by dense areas of fibers compared to the diffuse-porous structure in *U*. *californica* (Fig 1C–1E). This vascular arrangement could enhance redundancy to embolism formation within *Q*. *agrifolia*. Additionally, *Q*. *agrifolia* may already be so well adapted to drought stress that herbivore browsing may not significantly increase vascular stress from cavitation and embolism formation. Evidence of this drought stress tolerance can be seen in Fig 4, with *Q*. *agrifolia* showing no decline in Ψ_mid_ from early to late summer while *U*. *californica* shows a significant decline. Being extremely drought tolerant, *Q*. *agrifolia* may inherently possess the hydraulic adaptations predisposing it to herbivore browsing resistance that *U*. *californica* does not [43, 44], such as enhanced vascular recovery from cavitation and increased vessel redundancy.

While *U*. *californica* reduced conductive vessel density within browsed zones, it did not show a corresponding reduction in stomatal conductance or Ψ_mid_ for browsed stems in either early or late summer (Fig 4B and 4D; note Ψ_mid_ decline across season for both zones), nor did it show altered stem vessel dimensions. Rather, the reduction in leaf area appears to be the primary driver of the reduction in conductive stem vessels. This response can be explained by reduced water supply to developing leaves, reducing leaf expansion [45, 46]. Smaller leaves require less water to maintain turgor, allowing similar stomatal conductance rates as larger leaves and similar water potentials as stems from non-browsed zones. An important consequence of this reduced leaf size could be a net reduction in photosynthate produced, leading to a potential reduction in biomass accumulation for browsed zones [47].

An important factor in vascular development and function is rooting depth, which we did not include in our study due to COVID-related work interruptions. Evidence of hydraulic architecture in roots suggests that rooting depth is positively correlated with an increase in conduit hydraulic diameter and a more positive water potential in roots [48]. This likely impacts overall hydraulic architecture and water transport throughout the tree, potentially allowing for increased water and nutrients, which could offset the cost of producing new growth following herbivory. Since rooting depth for both *Q*. *agrifolia* and *U*. *californica* is unknown at this site, this trait could very well have impacted our results and warrants further investigation.

## Conclusion

The findings presented in this paper suggest that herbivory can be a complex moderator of leaf and stem adaptations, and species-specific responses play an important role. The ability of plants to respond to herbivores has been well documented, with notable examples of response to ungulate browsing [8, 10, 19]. However, the physiological and functional plant traits that herbivores impact can be species-specific rather than general across taxa [49]. Here we have shown a correlation between browsing and leaf architecture via leaf and stem vascular trait responses that appear species-dependent and coordinated with leaf development. We also found that browsing generally led to decreased xylem capacity, although by different pathways for each species. Most surprising was the coordination and scaling of leaf traits across leaf size instead of an alteration of anatomical structure between browsed and non-browsed zones, resulting in browsed leaves with stomata and epidermal cell traits comparable to non-browsed leaves. A robust species-specific response to herbivory appears to occur within this system, where browsing impacts functional traits in alternate ways across different species. Future studies should consider the species-specific responses to herbivory to help elucidate mechanisms that can truly be considered universal for coordinated trait development and which traits need to be considered independently. Additional research on the interplay of ecological factors beyond the scope of this study, such as multiple herbivore interactions and timing of budburst and herbivory on hormone effects, would further aid in describing plant trait responses to browsing under natural conditions [50–52].

## Supporting information

S1 Fig Browsed *Q*. *agrifolia* correlation plot. Pearson correlation values represent the degree of correlation between traits in matrix. Traits are arranged alphabetically along axes.
(TIF)

S2 Fig Non-browsed *Q*. *agrifolia* correlation plot. Pearson correlation values represent the degree of correlation between traits in matrix. Traits are arranged alphabetically along axes.
(TIF)

S3 Fig Browsed *U*. *californica* correlation plot. Pearson correlation values represent the degree of correlation between traits in matrix. Traits are arranged alphabetically along axes.
(TIF)

S4 Fig Non-browsed *U*. *californica* correlation plot. Pearson correlation values represent the degree of correlation between traits in matrix. Traits are arranged alphabetically along axes.
(TIF)

S1 Table Linear regression model results. Results from the lm() function in R for each trait pair displayed in Fig 3. Each cell shows the summary printout for browsed and non-browsed data sets. Cells are arranged to correspond to Fig 3, with 3A referring to *Q agrifolia* data sets and 3B referring to *U*. *californica* data sets.
(PDF)
