# Supplementary figures and images for: Species-specific responses drive browsing impacts on physiological and functional traits in Quercus agrifolia and Umbellularia californica
Source: PLoS One. 2024 Jul 24;19(7):e0287160. doi: 10.1371/journal.pone.0287160 (PMC11268663; doi:10.1371/journal.pone.0287160)

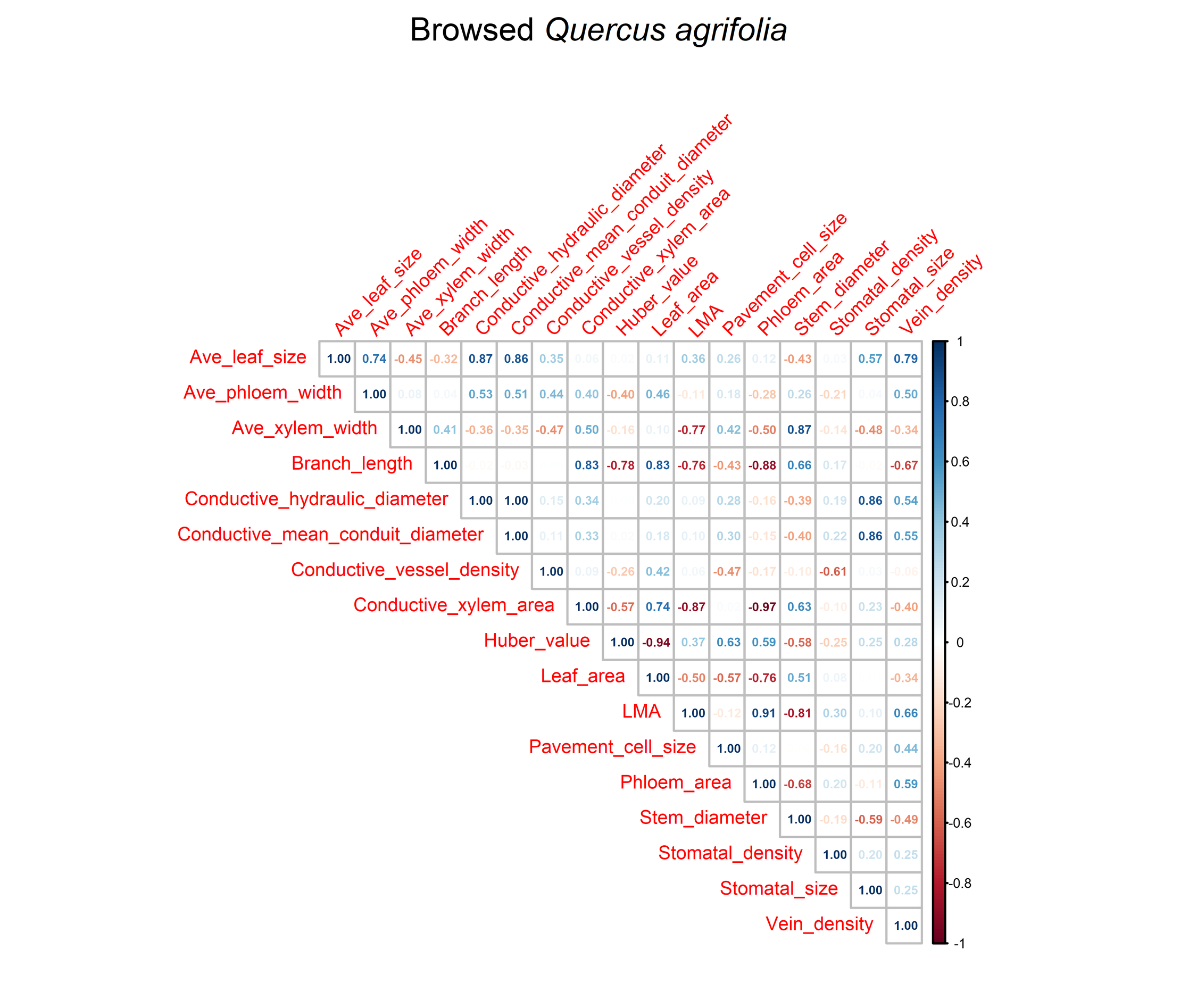

Supplement: S1 Fig — Pearson correlation values represent the degree of correlation between traits in matrix. Traits are arranged alphabetically along axes. (TIF) [file pone.0287160.s001.tif]

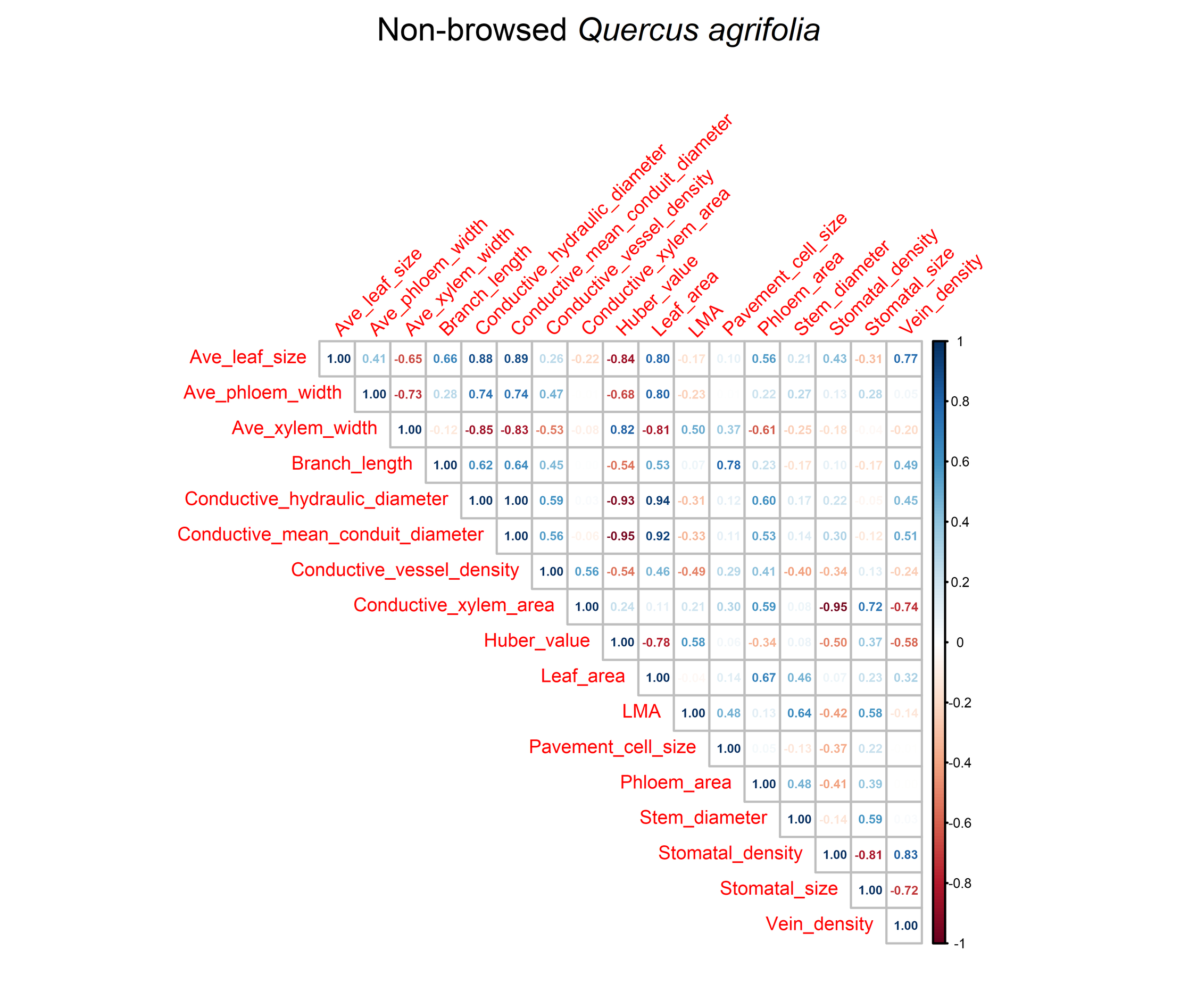

Supplement: S2 Fig — Pearson correlation values represent the degree of correlation between traits in matrix. Traits are arranged alphabetically along axes. (TIF) [file pone.0287160.s002.tif]

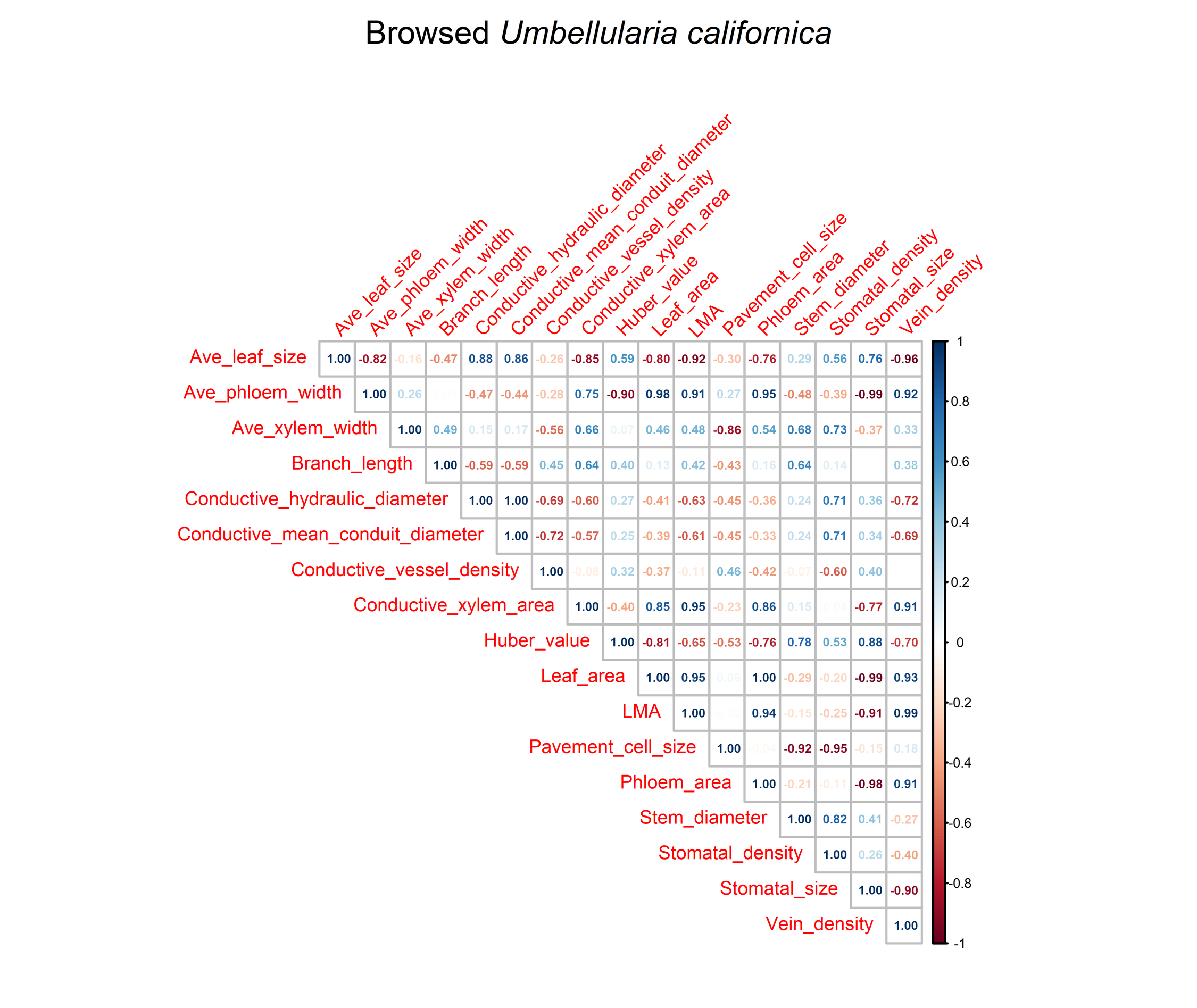

Supplement: S3 Fig — Pearson correlation values represent the degree of correlation between traits in matrix. Traits are arranged alphabetically along axes. (TIF) [file pone.0287160.s003.tif]

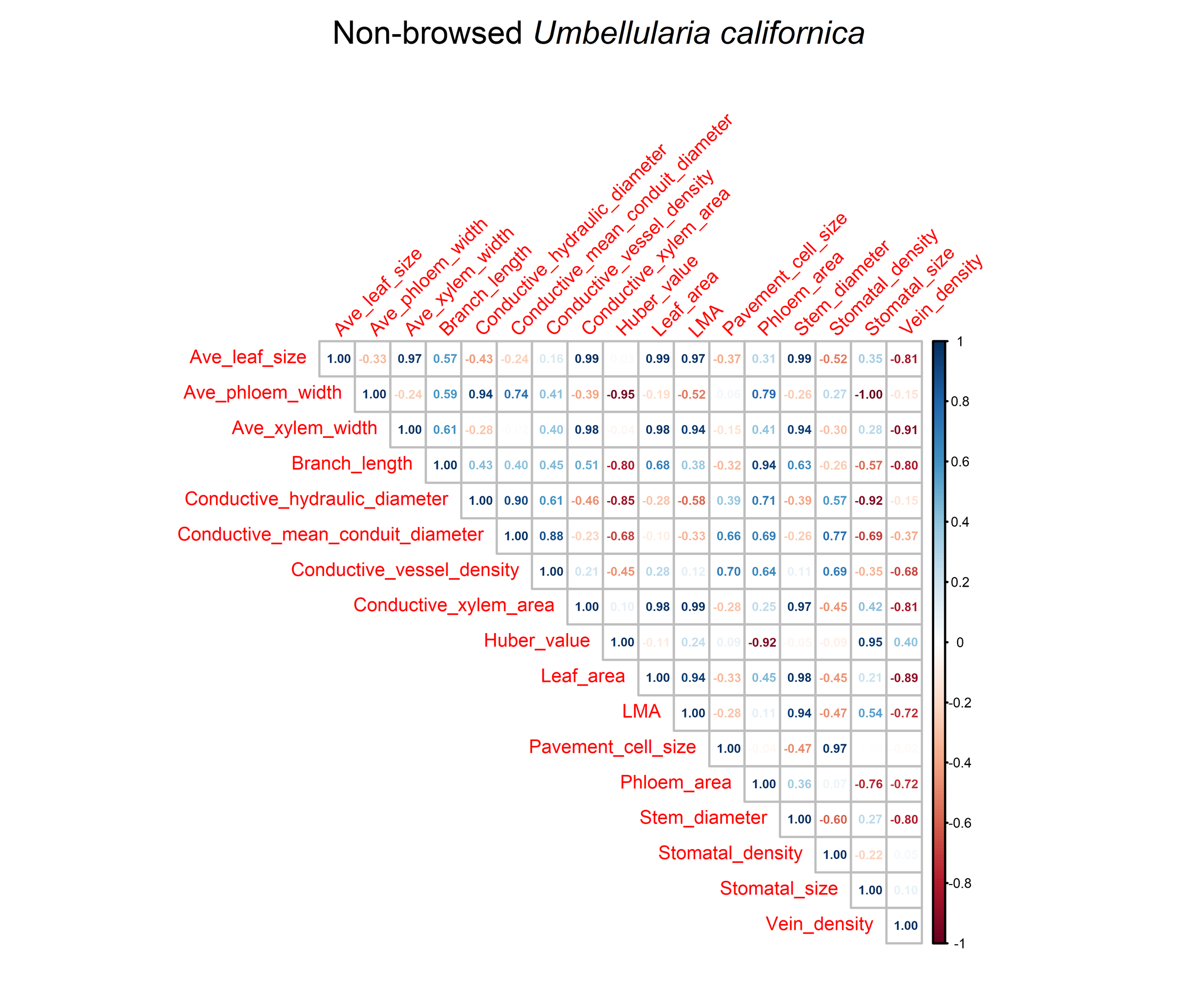

Supplement: S4 Fig — Pearson correlation values represent the degree of correlation between traits in matrix. Traits are arranged alphabetically along axes. (TIF) [file pone.0287160.s004.tif]
