# Supplementary material for: Species-specific responses drive browsing impacts on physiological and functional traits in Quercus agrifolia and Umbellularia californica
Source: PLoS One. 2024 Jul 24;19(7):e0287160. doi: 10.1371/journal.pone.0287160 (PMC11268663; doi:10.1371/journal.pone.0287160)
Supplement: S1 Table — Results from the lm() function in R for each trait pair displayed in Fig 3. Each cell shows the summary printout for browsed and non-browsed data sets. Cells are arranged to correspond to Fig 3, with 3A referring to Q agrifolia data sets and 3B referring to U. californica data sets. (PDF) [file pone.0287160.s005.pdf]

S1 Table: Linear regression model results for browsed and non-browsed data sets within *Q. agrifolia* and *U. californica*.

Fig 3A: linear regression model for browsed zone *Q. agrifolia* data

```
Call:
lm(formula = stomatal_density_per_mm.2 ~ epidermal_size_um.2,
    data = qb)

Residuals:
     Min       1Q   Median       3Q      Max
-112.235  -14.458   -1.422   31.465   95.786

Coefficients:
                    Estimate Std. Error t value Pr(>|t|)
(Intercept)         663.9901   123.1288   5.393 0.000652 ***
epidermal_size_um.2  -1.1333     0.5612  -2.020 0.078100 .
---
Signif. codes:  0 ‘***’ 0.001 ‘**’ 0.01 ‘*’ 0.05 ‘.’ 0.1 ‘ ’ 1

Residual standard error: 71.19 on 8 degrees of freedom
Multiple R-squared:  0.3377,  Adjusted R-squared:  0.2549
F-statistic: 4.079 on 1 and 8 DF,  p-value: 0.0781
```

Fig 3A: linear regression model for non-browsed *Q. agrifolia* data

```
Call:
lm(formula = stomatal_density_per_mm.2 ~ epidermal_size_um.2,
    data = qnb)

Residuals:
     Min       1Q   Median       3Q      Max
-105.584  -44.292   -3.405   49.515  114.475

Coefficients:
                    Estimate Std. Error t value Pr(>|t|)
(Intercept)         827.3680   128.6085   6.433 0.000202 ***
epidermal_size_um.2  -1.9126     0.6612  -2.893 0.020116 *
---
Signif. codes:  0 ‘***’ 0.001 ‘**’ 0.01 ‘*’ 0.05 ‘.’ 0.1 ‘ ’ 1

Residual standard error: 75.41 on 8 degrees of freedom
Multiple R-squared:  0.5112,  Adjusted R-squared:  0.4501
F-statistic: 8.368 on 1 and 8 DF,  p-value: 0.02012
```

Fig 3B: linear regression model for browsed *U. californica* data

```
Call:
lm(formula = stomatal_density_per_mm.2 ~ epidermal_size_um.2,
    data = ub)

Residuals:
    Min      1Q  Median      3Q     Max
-51.913 -26.578  -1.352  29.703  58.687

Coefficients:
                     Estimate Std. Error t value Pr(>|t|)
(Intercept)         404.93403   48.01653   8.433 2.98e-05 ***
epidermal_size_um.2  -0.35724    0.08664  -4.123  0.00333 **
---
Signif. codes:  0 ‘***’ 0.001 ‘**’ 0.01 ‘*’ 0.05 ‘.’ 0.1 ‘ ’ 1

Residual standard error: 39.89 on 8 degrees of freedom
Multiple R-squared:   0.68,     Adjusted R-squared:   0.64
F-statistic:    17 on 1 and 8 DF,  p-value: 0.00333
```

Fib 3B: linear regression model for non-browsed *U. californica* data

```
Call:
lm(formula = stomatal_density_per_mm.2 ~ epidermal_size_um.2,
    data = unb)

Residuals:
   Min     1Q Median     3Q    Max
-52.64 -31.00 -11.33  29.62  74.38

Coefficients:
                    Estimate Std. Error t value Pr(>|t|)
(Intercept)         304.8353    65.1705   4.678  0.00159 **
epidermal_size_um.2  -0.2055     0.1296  -1.586  0.15134
---
Signif. codes:  0 ‘***’ 0.001 ‘**’ 0.01 ‘*’ 0.05 ‘.’ 0.1 ‘ ’ 1

Residual standard error: 45.13 on 8 degrees of freedom
Multiple R-squared:  0.2393,  Adjusted R-squared:  0.1442
F-statistic: 2.516 on 1 and 8 DF,  p-value: 0.1513
```

Table arranges the models in order of browsed and non-browsed data sets for each species.
